# Analysis of the dynamics of limb transcriptomes during mouse development

**DOI:** 10.1186/1471-213X-11-47

**Published:** 2011-07-29

**Authors:** Istvan Gyurján, Bernhard Sonderegger, Felix Naef, Denis Duboule

**Affiliations:** 1 School of Life Sciences, Ecole Polytechnique Fédérale, Station 19, Lausanne, 1215 Switzerland; 2 Swiss Institute of Bioinformatics, Batiment Génopode, University of Lausanne, 1215 Switzerland; 3 Department of Genetics and Evolution, University of Geneva Quai Ernest Ansermet, 30, Geneva, 1211 Switzerland

## Abstract

**Background:**

The development of vertebrate limbs has been a traditional system to study fundamental processes at work during ontogenesis, such as the establishment of spatial cellular coordinates, the effect of diffusible morphogenetic molecules or the translation between gene activity and morphogenesis. In addition, limbs are amongst the first targets of malformations in human and they display a huge realm of evolutionary variations within tetrapods, which make them a paradigm to study the regulatory genome.

**Results:**

As a reference resource for future biochemical and genetic analyses, we used genome-wide tiling arrays to establish the transcriptomes of mouse limb buds at three different stages, during which major developmental events take place. We compare the three time-points and discuss some aspects of these datasets, for instance related to transcriptome dynamics or to the potential association between active genes and the distribution of intergenic transcriptional activity.

**Conclusions:**

These datasets provide a valuable resource, either for research projects involving gene expression and regulation in developing mouse limbs, or as examples of tissue-specific, genome-wide transcriptional activities.

## Background

Limb development has fascinated biologists for a century, mostly because of the importance of these structures in the evolution of land vertebrates and due to their spectacular morphological diversity. From an experimental viewpoint, limbs are quite easily accessible at various stages of their ontogenesis and can thus be manipulated, to some extent. Genetically speaking, limb phenotypes can be easily detected and usually do not impair survival too strongly. For all these reasons, limbs have been excellent model structures to study vertebrate patterning and morphogenesis.

Tetrapods limbs bud out from the lateral plate mesoderm and establish early on the bases of a three-dimensional pattern. The growth along the proximo-distal axis largely depends on FGFs signaling emanating from the apical ectodermal ridge (AER) and acting over the mesenchyme. On the other hand, the anterior to posterior (AP) axis is specified essentially by *Hand2*, the *Shh *pathway and posterior *Hoxd *genes. Amongst the known regulators of the dorso-ventral (DV) patterning are *Wnt7a*, expressed in dorsal ectoderm, and *Engrailed*, in ventral ectoderm, as well as *Lmxb1*, a gene transcribed in dorsal mesenchyme. These signaling cascades act together in a highly coordinated manner [1-3].

At later stages of development, *e.g*. starting from E11.5 onwards in mice, mesenchymal condensations form and establish the future skeletal pattern, following a tightly regulated process. The first elements to appear are those of the future stylopod (humerus, femur), then of the zeugopod (radius, ulna, tibia, fibula) and, finally, the autopod (hands and feet). This period of limb development, referred to as 'proliferative expansion, determination and differentiation' [2], is a very dynamic phase, where differences amongst tetrapods start to be translated from the genetics (the transcription, maintenance and silencing of regulatory genes and their readouts) to species-specific morphologies. Such differences in the shapes of tetrapod limbs, may rely upon slight variations in these 'genetic' parameters, such as transcriptional heterochronies or quantitative effects of key developmental molecules [4-6].

While many of those genes critical for limb development were described over the past 15 years, additional players, such as regulatory RNAs, have come into play more recently. There is indeed increasing evidence that long non-coding RNAs (ncRNAs) may be crucial for some developmental processes [7] due to their involvement in various functions including gene regulation, both at the transcriptional and post-transcriptional levels, in the organization of epigenetic modifications [8] as well as during gene activation and silencing [9]. Surprisingly, inter-species sequence conservation does not seem to be essential [10], even in the cases of the *Xist, Air *or *Evf-2 *RNAs where important functions were demonstrated [11-13]. The best-studied long ncRNAs are associated with imprinted gene clusters, where many of them act by repressing neighboring genes *via *a *cis*-effect [13,14].

As a resource for research projects involving gene expression and regulation in developing mouse limbs, we set up to produce and analyze whole genome expression data for E11.5, E13.5 and E15.5 forelimbs. We selected these three stages of embryonic limb development not only because these days are very dynamic, in terms of growth and organogenesis, but also because the transcriptional activation of several known genes occurs during this time-period, giving us both an idea of the relevance of the datasets, and the possibility to look at the vicinities of these loci to evaluate a potential clustering of transcriptional activities. Within this time-window indeed, the muscular and skeletal systems develop in parallel with massive vascularization, innervations and skin formation, implying large amounts of new cell types and tissue interactions. We selected three different ontogenetic stages to see how global transcriptional activity evolves along with development of a complex structure, not only by considering protein coding genes, but also intergenic transcription and non-coding RNA expression.

## Results and discussion

**Figure 1:**
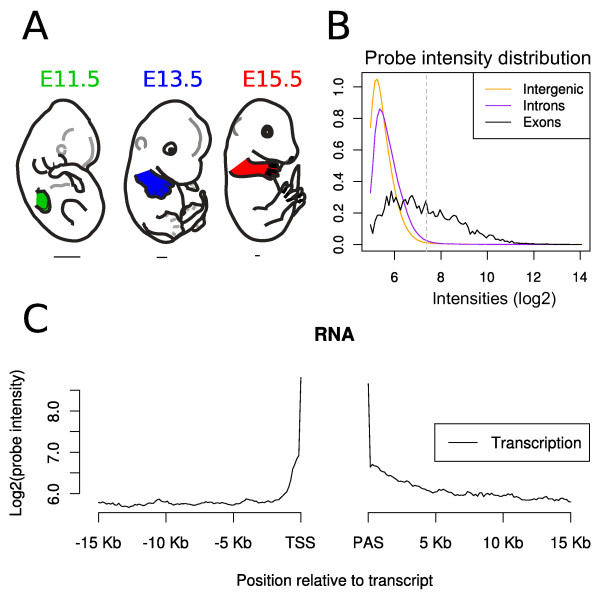
**Experimental setup and tiling array intensity distributions**. **A: **Schematics of embryos at the three stages of limb development (E11.5, E13.5 and E15.5) considered in this study. Forelimbs are indicated with a color code that is used in subsequent figures. The bars below the embryos indicate relative size differences. **B: **Histogram showing the intensity distributions along chromosome 2 for intergenic, intronic and exonic transcription. Exons considered are as defined in UCSC Known Genes annotation. **C: **Distribution of probe intensity around expressed genes on chromosome 2. TSS: transcription start site; PAS: polyadenylation site. Vertical gray dotted line: determined threshold level (in this example log2 = 7.554).

We sampled mouse embryonic forelimbs at days 11.5, 13.5 and 15.5 postcoïtum, by cutting off the entire forelimb buds from the body wall (Figure 1A). The particular morphogenetic events occurring at these stages have been largely described earlier (*e.g. *[15]). Briefly, E11.5 limb buds consist of a rather poorly differentiated inner mesenchyme, located within an epidermal envelope. Despite this apparent uniformity, mesenchymal cells at this stage can be already differentiated as belonging to denser, centrally located regions where condensation of the future cartilage rods starts to occur [2]. These condensations appear with a time sequence that follows a proximal to distal progression. At the same stage, the first nerve fascicles become apparent, originating from the brachial plexus.

By day 13.5, the cartilage primordia of the future limb skeleton are already well defined, the digits start to separate from one another as a result of inter-digital cell death. There is massive deposition of muscle elements (already initiating in late E11.5 buds), nerves extend into the hand plates and few hair buds are already apparent. The limbs are fully vascularised and the skin starts to stratify. Embryonic forelimbs at day 15.5 have completely separated digits. The ossification process, which starts around E14.0, is in progress even in the most distal phalanges. Most of the tendons are now well defined and there is strong progression of skin stratification.

### Expression signals are frequently confined to known transcript boundaries but tend to extend past the 3'ends of genes

Following RNA extraction and processing (see materials and methods) labelled cDNAs were hybridized onto whole genome tiling arrays (Affymetrix, Genechip Mouse Tiling 1.1R). After data normalization (see materials and methods) we used the UCSC Known Gene annotation system (mm9, 2007) to quantify expression levels in exons, introns and intergenic regions. Intensity thresholds to determine expressed transcripts and genes were calculated for each array, as exemplified by using chromosome 2 (Figure 1B). Distributions showed low intensities both in introns and intergenic regions, with introns showing slightly higher signals possibly reflecting precursor mRNAs or splice forms not covered by our annotation. In contrast the distributions for exons were clearly shifted towards higher expression. We also observed a bias in intensity around annotated transcripts since the signals in regions downstream of the annotated transcript ends were usually more pronounced and decayed more slowly than in regions upstream of the transcription start sites (TSSs). Also, in several cases, the 3' untranslated regions (UTR) were much longer than anticipated from the UCSC Known Gene annotation (Figure 1C). Overall, we observed that transcription was mostly confined to the vicinity of annotated genes, a finding that is consistent with RNA-seq studies in mouse and human [16], though some novel transcription sites were found (see below).

### About 40% of known genes are expressed at E11.5, E13.5 and E15.5

**Figure 2:**
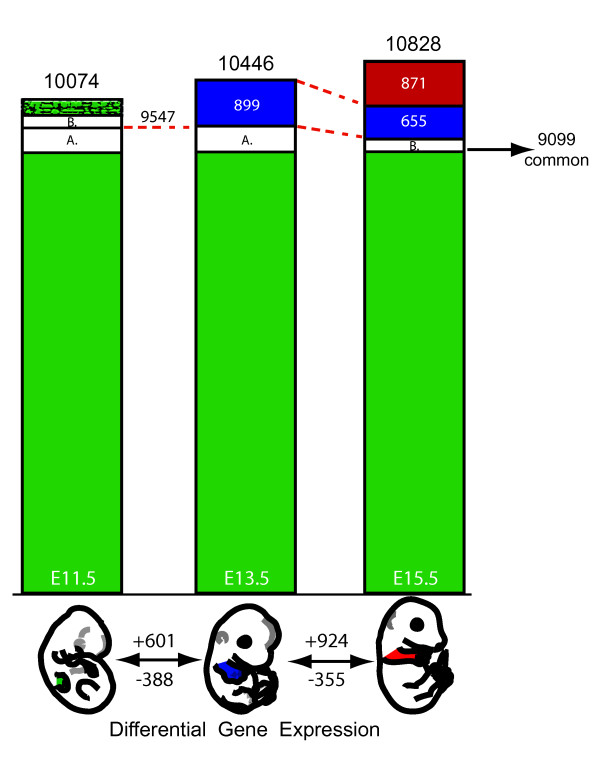
**Gene expression dynamics during three stages of limb development**. **Top: **The expressed UCSC Known Genes at the three limb developmental stages. Smooth green areas represent genes expressed at all three stages (n = 9099). Blue areas show genes whose transcription is gained at E13.5 (n = 899) and still found expressed in embryos at E15.5 (n = 655). The red area represents genes that are significantly expressed only at E15.5 (n = 871). The patterned green area in E11.5 highlights those genes that are expressed only at E11.5 (n = 297). The white areas indicate genes that are expressed in both E11.5 and E13.5 limbs, but not at E15.5 (**A**; n = 475), or with genes expressed in both E11.5 and E15.5 (**B**; n = 203), yet not in E13.5 limbs. **Bottom: **Differential gene expression in forelimbs between the developmental stages. The numbers of up- and down-regulated genes are indicated above and below the arrows, respectively.

The global expression data for individualized exons, transcripts and genes at the three developmental stages are shown in Table 1 (panel A). 41.17%, 42.69 and 44.25% of known genes were expressed in forelimbs at E11.5, E13.5 and E15.5, respectively. Based on UCSC gene models, about 90% of these were annotated as coding genes (we incorporated 'near-coding genes' into this category). Interestingly about 95% of all expressed genes were common to the three developmental stages, with an average increase of 4% between E11.5 towards E15.5 limbs (Figure 2).

**Table 1:** Summary of gene expressed during the development of mouse embryonic limb at the three developmental stages studied

Developmental stage			*E.11.5*	*E13.5*	*E15.5*
**A**	UCSC known gene	**Exons**	45.45% (102020/224464)	47.01% (105530/224464)	48.65% (109203/224464)
		**Transcripts**	24.91% (11500/46163)	25.99% (12000/46163)	26.23% (12113/46163)
		**Genes**	41.17% (10074/24469)	42.69% (10446/24469)	44.25% (10828/24469)
	
	Gene models	Coding	90.71% (9139/10074)	90.5% (9454/10446)	91.35% (9892/10828)
		Antisense	2.29% (231/10074)	2.42% (253/10446)	2.25% (244/10828)
		Non-coding	6.98% (704/10074)	7.07% (739/10446)	6.39% (692/10828)

**B**	Genes with intronic signal (if the gene is expressed)		4841 (3296)	5155 (3662)	4660 (3259)

**C**	Intergenic regions		7408	7471	7477
	
	Directly flanking UCSC genes		528 (7.13%)	526 (7.04%)	537 (7.18%)
	Identifiable via ENSEMBL		1981 (26.74%)	1970 (26.37%)	1994 (26.67%)
	Directly flanking ENSEMBL genes		587 (7.92%)	576 (7.71%)	592 (7.92%)
	Unexplained		4312 (58.21%)	4399 (58.88%)	4354 (58.23%)

**A: **UCSC Known Gene expression and gene model categories. **B: **Intronic transcription. Numbers in parentheses shows those intronic transcripts for which the genes containing them were also expressed. **C: **Transcription of intergenic regions and sub-categories (see Materials and Methods). The 'unexplained' category represents transcribed intergenic regions remaining after filtering (cf. Methods).

### Differential gene expression

We compared the expressed genes between the three developmental stages to evaluate which percentage was significantly changed along with limb development (see Methods). Between E11.5 and E13.5, we identified 601 up-regulated genes and 388 down-regulated genes (Figure 2, Additional file 1). Between E13.5 and E15.5 forelimbs, 924 genes were found up-regulated and 355 down-regulated. While these figures indicate that the general tendency goes towards an increase in the number of genes expressed, along with developmental time, they should be considered carefully since they derive from comparisons between steady-state levels of RNAs. Consequently, they do not necessarily reflect transcriptional switches and may point to RNAs whose transcription has increased, in the case of a gene considered as 'newly expressed'. Likewise, RNA stability may prevent, in some cases, the timely detection of a particular transcriptional switch-off. In addition, the cellular topographies of gene expression patterns are usually not homogenous and hence variations in relative transcript abundance may also indicate variations in space rather than in time (see below).

We looked into these parameters by quantitative PCRs on some of the genes, for which changes in expression patterns had been previously reported (Additional file 2). In addition, six genes with either known or unknown expression profiles, were validated by *in situ *hybridization (Additional file 2). As examples, the *Cbln1 *mRNA, essential during synapse formation [17] yet with previously unknown profile, was weakly expressed at E11.5 only, whereas the *Tmem8c *gene was specifically expressed in early muscle elements along both the trunk and the limb and progressively increased its expression throughout development.

To evaluate whether differentially expressed genes could be grouped into particular functional categories, we used the Ingenuity Knowledge Base http://www.ingenuity.com. This analysis indicated that functional categories such as muscle and skeletal system development, or skin development were enriched, as expected from the developmental stages under scrutiny (Table 2). We also performed a more detailed gene ontology analyses using GOMiner [18], to see if development related terms were enriched in our sets of differentially expressed genes. Functions were clustered into hierarchical trees (see Additional files 3 and 4) and the results showed that terms related to muscle development again dominated, both when E11.5 limbs were compared with E13.5, and when E13.5 limbs were compared with E15.5. This is most likely due to high number of genes encoding muscle structural proteins. Cartilage, bone, and cell-adhesion related term enrichments were also scored. Noteworthy, the number of functional clusters was higher between E13.5 and E15.5 than between the two younger stages, which reflects an increased tissue diversification along with the processing of limb development. We matched our sets of differentially expressed genes with the mammalian phenotype database http://www.informatics.jax.org and scored 69 genes that had been previously associated with abnormal or short limb morphology (Additional file 5).

**Table 2:** Ingenuity top five categories of differentially expressed genes concerning the development of physiological system

Differential Gene Expression: E13,5 *vs*. E11,5		
Name	p-value*	Number of Molecules
Tissue Development	6,56E-14 - 4,42E-03	148
Skeletal and Muscular System Development and Function	4,51E-11 - 4,42E-03	160
Organ Development	4,87E-11 - 4,03E-03	137
Cardiovascular System Development and Function	1,35E-08 - 4,42E-03	92
Tissue Morphology	9,38E-08 - 4,42E-03	138

**Differential Gene Expression: E15,5 *vs*. E13,5**		
**Name**	p-value*	**Number of Molecules**

Skeletal and Muscular System Development and Function	2,06E-19 - 1,45E-03	239
Tissue Morphology	2,06E-19 - 1,13E-03	217
Tissue Development	6,00E-16 - 1,42E-03	283
Hematological System Development and Function	2,63E-11 - 1,35E-03	205
Hair and Skin Development and Function	3,28E-11 - 7,80E-03	74

*Fisher's exact tests were used to calculate p-values.

### Intronic and intergenic transcription

Although we applied a rather stringent threshold, we observed extensive intronic transcription, since about 50 percent of expressed genes also contained signals (≥300 bp) covering intronic sequences (Table 1B). While, in some instances, this can be explained by introns retained after splicing, the majority of this intronic activity seems to derive either from alternative start sites, as suggested by looking at some profiles of histone post-translational modification tracks from various cell types in the UCSC Genome Browser [19,20], or from alternative transcription termination, which seems to occur frequently in human or mouse genes and can thus lead to intronic transcription [21]. In addition, about 30 percent of these intronic signals were detected within genes that are not expressed during limb development, indicating the presence of independent transcription units overlapping with known genes.

We also scored 7408, 7471 and 7477 transcribed regions above threshold (≥300 bp), at E11.5, E13.5 and E15.5, respectively, within intergenic regions (Table 1C). We further filtered this raw data using the Ensembl database for possible overlaps and approximately 25 percent of these sequences were thus identified. Half of these matched protein coding genes, whereas the other half was composed of pseudogenes and retrotransposed elements (Additional file 6). Protein coding genes showed 48 percent parity with UCSC coding genes which, due to different annotation of 3'UTR sequences, and hence because of their different lengths, had not been identified earlier. After this filtering, 4312, 4399 and 4354 intergenic regions remained clearly transcribed during the three developmental stages, respectively (hereafter termed the 'unexplained' category). Some of these regions may reflect alternative start sites, extended 3'UTRs, retrotransposed or pseudogene elements, as well as non-coding RNAs.

**Figure 3:**
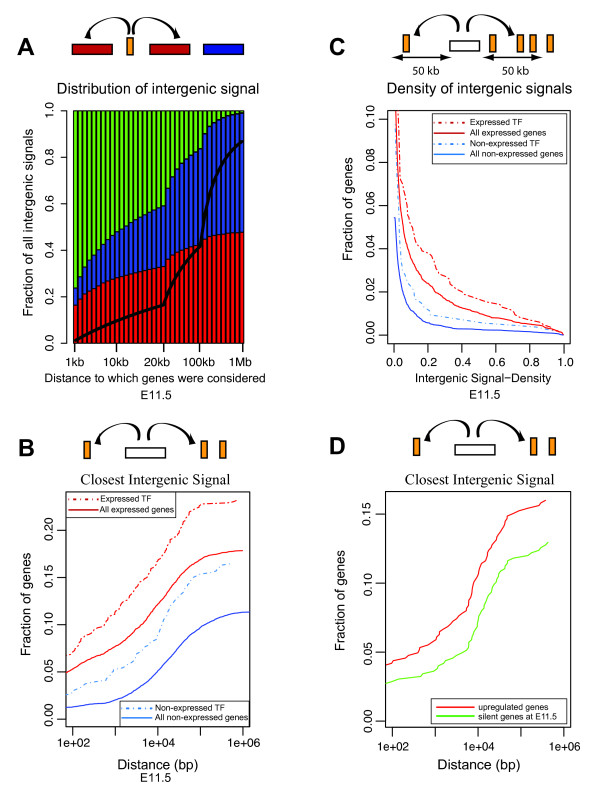
**Intergenic transcription and proximity to expressed genes**. **A: **All 4312 expressed intergenic regions (orange) at E11.5 were annotated as 'close to' an expressed gene (red), 'close to' a non-expressed (blue), 'not close to' a gene (green). Black lines show the expected borders between blue and green areas if genes were distributed randomly relative to expressed intergenic regions. **B: **Expressed genes are enriched with nearby intergenic transcription. Fraction of genes (expressed, solid red line N = 8582; or not-expressed, solid blue line N = 14848) with an expressed intergenic region within x bp is plotted over genomic distance (x-axis, log scale). Transcription factors (TF) selected based on Gene Ontology terms (GO:0003700; GO:0006355) (expressed, dashed red line N = 847; not expressed, dashed blue line N = 534) showed higher fractions in both categories when compared to all genes. **C: **A gene neighborhood was defined as its upstream and downstream regions half-way to the next annotated gene or maximally 50 Kb. The fraction of genes (expressed, red; not-expressed, blue), which contain unexplained intergenic signals within neighborhood is shown. Expressed genes have more of their neighborhoods covered by intergenic signals (top red and blue line). **D: **Transcription of intergenic sequences increases as nearby gene expression increases. For a selection of genes (N = 525) up-regulated from below threshold at E11.5, to above threshold at E13.5 or E15.5, the fraction with nearby intergenic signals is shown as in B at E11.5 (green) and E15.5 (red). A higher fraction of genes with nearby intergenic transcription is seen at E15.5, as the expression increases.

We next asked whether these transcribed intergenic regions were distributed randomly throughout the genome or, alternatively, whether they would tend to be associated with (or be located at the vicinity of-) active (or silent) gene loci. We thus analyzed the transcriptional status of those genes located around each transcribed intergenic region, to assess whether they were themselves expressed or not (Figure 3). Our genome-wide survey indicates that transcribed 'intergenic regions' are indeed found more frequently at the vicinity of transcribed genes, an observation valid as long as a distance of less than about 50 kb is considered. If a larger distance is allowed, this association becomes less significant (Figure 3A). The same tendency was observed when we estimated the fraction of expressed, or non-expressed genes, with flanking transcribed intergenic regions (Figure 3B-D). There again, a higher fraction of expressed genes, as compared to non-expressed, showed flanking intergenic transcription. In this case, interestingly, this correlation was increased when considering the sub-group of genes coding for transcription factors (Figure 3B,C) and even non-expressed transcription factor-coding genes appeared to be more frequently surrounded by transcribed intergenic regions.

These results may reflect the presence, in the vicinity of annotated genes, of alternatively spliced regions, or alternate start sites, located at a distance, which had not been previously identified. It may also indicate that intergenic transcription may result from the nearby transcription of a 'standard' transcription unit, *via *a generic by-stander effect. For example, modifications of the epigenetic landscape associated with gene transcription may generate ectopic transcripts in the neighbourhood. In support of this, the fact that this observation is accentuated around transcription factors, *i.e. *nearby genes that, at least during development, are often controlled by remote regulations, over large distances [22]. We also measured the frequency of intergenic signals around those genes up-regulated between E11.5 and E15.5. However, the number of such genes with associated intergenic signals within a given distance was too low to be fully significant. The fact that transcription factor genes scored as 'not-expressed' showed an above-average occurrence of intergenic signal around them raises the possibility that these loci may maintain 'open' local chromosomal domains for subsequent activation, as proposed in the case of embryonic stem (ES) cells based on the existence of a bivalent state in chromatin modifications [23].

### Differentially expressed ncRNAs

We searched for a differential expression of non-coding RNAs (ncRNAs) annotated in UCSC, between the three developmental stages. After careful (manual) curation, which removed all potential 3'UTRs, pseudogenes and non-coding isoforms of coding genes, we identified 49 long ncRNAs that were differentially expressed in our samples (Table 3). Amongst them, ten were typical bidirectional transcripts, one was a *cis*-antisense transcript, two were antisense in the 3' end of genes (tail-to-tail), whereas the remaining ncRNAs were not closely associated with any gene. Twenty-two ncRNAs increased their relative amounts at steady state levels, between E11.5 and E13.5, whereas eleven of them decreased in amount between E11.5 and E13.5. Between E13.5 and E15.5, eleven ncRNAs were up-regulated and 25 down-regulated. As an example, the ncRNA *Neat1 *appeared as progressively up-regulated, consistent with the earlier observation that *Neat1 *expression was re-enforced during muscle cell differentiation [24]. We also assessed the level of conservation of these transcripts (using PhasCons scores) and found that they were globally poorly conserved in sequences amongst vertebrates, in agreement with the idea that the transcription itself of these RNAs or their structure, rather than sequence conservation, may be important [25,10].

**Table 3:** List of differentially expressed long non-coding RNAs, as annotated in UCSC.

Gene Symbol	UCSC ID	Location	PhasCons Score (mean)	Orientation to the closest coding gene	Closest Coding Gene	Differential Gene Expression	
						E13.5 vs E11.5	E15.5 vs E13.5
AK019125	uc007byu.1	Chr1	0.08663	head-to-head	Arl4c	1	-1
AK003315 (Myeov2)	uc007cbq.1	Chr1	0.05824	head-to-tail	Otos	-1	0
AK014513	uc007eem.1	Chr1	0.10996	tail-to-head	Camk1g	1	1
AK082757	uc008iue.1	Chr2	0.06682	tail-to-head	Btbd14a	0	1
AK007459	uc008iuf.1	Chr2	0.27284	head-to-head	Btbd14a	0	1
C130021l20Rik	uc008jhz.1	Chr2	0.13674	head-to-head	Lmx1b	0	-1
AK030275	uc008ltx.1	Chr2	0.13798	head-to-tail	Sid470	0	-1
BC048556	uc008mgc.1	Chr2	0.21847	tail-to-head	Nphp1	1	1
AK007971	uc008nqb.1	Chr2	0.09166	tail-to-tail	Lbp	0	-1
AK012506	uc008onz.1	Chr3	0.34076	antisense	Zfhx4	0	-1
AK007373	uc008rbp.1	Chr3	0.09312	head-to-head	Dph5	-1	0
BC096391 (Snhg8)	uc008rfm.1	Chr3	0.20019	tail-to-tail	Prss12	0	-1
AK080292	uc008rzc.1	Chr4	0.17915	head-to-head	Ccne2	0	-1
NR_003270 (Snhg3)	uc008vbf.1	Chr4	0.33261	head-to-tail	Phactr4	-1	-1
AK083203	uc008wbz.1	Chr4	0.09120	haid-to-head	Prdm16	0	-1
AK012278	uc008xth.1	Chr5	0.22535	head-to-head	Usp46	-1	0
2610001J05Rik	uc009ayt.1	Chr6	0.13108	tail-to-head	Gpr85	1	0
AK002748	uc009bxj.1	Chr6	0.05646	head-to-head	Nfe2l3	1	0
AK033508	uc009byt.1	Chr6	0.19038	head-to-head	Hoxa13	-1	-1
AK039589	uc009cib.1	Chr6	0.08391	head-to-tail	St3gal5	1	-1
AK170805	uc009ejz.1	Chr6	0.11942	head-to-head	Tas2r140	1	0
AK011885	uc009ely.1	Chr6	0.28077	head-to-head	Atf7ip	1	0
H19	uc009kob.1	Chr7	0.25529	tail-to-tail	Mrpl23	-1	1
AK134636	uc009las.1	Chr8	0.13898	tail-to-head	4930467E23Rik	1	0
Phxr4	uc009odu.1	Chr9	0.09209	tail-to-head	Maml2	1	0
BC003348	uc009oek.1	Chr9	0.66034	head-to-tail	Sesn3	0	-1
AK165129	uc007htj.1	Chr11	0.37820	head-to-head	Morc2a	1	-1
AK162965	uc007jdh.1	Chr11	0.18394	head-to-head	Mrp155	1	-1
AK079857	uc007kol.1	Chr11	0.06137	head-to-tail	Slfn3	1	-1
BC037520	uc007mmx.1	Chr11	0.10152	tail-to-head	Sec14l1	1	1
AK164256	uc007msb.1	Chr11	0.19866	head-to-head	Bahcc1	0	-1
1700012B15Rik	uc007mwg.1	Chr12	0.64135	tail-to-tail	Rab10	0	1
AK035058	uc007nvz.1	Chr12	0.30950	tail-to-tail	Six1	0	-1
AF498300 (Rian)	uc007paz.1	Chr12	0.05280	head-to-head	Rtl1	1	1
BC025054	uc007pyi.1	Chr13	0.11581	head-to-tail	Sox4	1	-1
AK087718	uc007pip.1	Chr13	0.15192	tail-to-head	Gdi2	-1	-1
AK021143	uc007qip.1	Chr13	0.06779	head-to-head	Fam120a	1	-1
AK020502	uc007qlx.1	Chr13	0.09589	head-to-head	Spin1	0	-1
AK029385	uc007vaf.1	Chr14	0.20634	tail-to-head	Slc15a1	-1	0
AK011684	uc007vbc.1	Chr14	0.25107	head-to-head	Zic2	-1	0
DQ715667	uc007wbs.1	Chr15	0.44104	tail-to-tail	Chrac1	-1	1
AK085438	uc007wpq.1	Chr15	0.10360	tail-to-head	Rac2	1	-1
AK005956	uc007zwv.1	Chr16	0.13992	head-to-head	1110004E09Rik	-1	1
AK004150	uc008cek.1	Chr17	0.09964	head-to-tail	Hspa1b	0	-1
Malat1	uc008gfj.1	Chr19	0.34059	tail-to-head	Scyl1	1	0
Neat1	uc008gfk.1	Chr19	0.18959	head-to-tail	Frmd8	1	1
AK051045	uc008gmk.1	Chr19	0.23573	head-to-head	Slc3a2	0	-1
AK030946	uc009tdn.1	ChrX	0.04767	head-to-tail	2610018G03Rik	1	-1
AK008724	uc009ura.1	ChrX	0.21428	head-to-head	Kctd12b	1	0

'1' means that the lincRNA is upregulated between the two stages considered, whereas '-1' indicated a down-regulation. The orientations with respect to the closest coding genes, as indicated, were not related to the distance between them. Conservation scores (PhasCons) indicate mean values, as determined from the alignments of 29 vertebrate genomes with the mouse (downloaded from UCSC).

**Figure 4:**
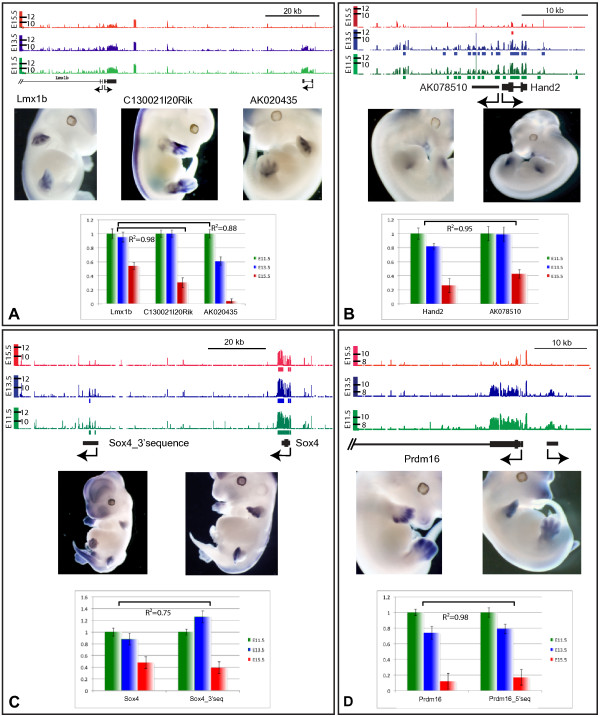
***In situ *hybridizations of selected intergenic transcripts**. The charts show relative amounts of transcripts as measured by qPCR at E11.5 (green), E13.5 (blue) or E15.5 (red). Expression levels at day E11.5 are normalized to 1. Correlation coefficients (R^2^) indicate the relative correlations between the expression of both the protein coding genes and the associated intergenic transcripts. Tiling array data (scales in log_2 _on Y-axes) and genomic maps are also shown. **A**. *Lmx1b *gene and associated upstream region, with bidirectional transcripts annotated here as *Lmx1b *and *C130021l20Rik*. A potential alternative start site for *Lmx1b*, 60 kb upstream (*AK020435*) shows the same expression specificity. **B**. Bidirectional transcription at the *Hand2 *locus, with both *Hand2 *and the *AK078510 *transcript displaying related patterns in the posterior margin of developing forelimbs. **C**. An as yet unknown intergenic transcript (*BC025054*) located 50 kb downstream of the *Sox4 *locus, on the same strand of chromosome 13, which is expressed like *Sox4*, suggesting it may be a remote 3' exon of this gene. **D: **Bidirectional transcription at the *Prdm16 *locus on chromosome 4, with an as yet unknown transcript mimicking *Prdm16 *expression in the E12.5 digital plate.

The expression of four long ncRNAs was examined in some details by *in situ *hybridization (Figure 4), both to validate the transcriptome data and because these ncRNAs are localised at the vicinity of genes known to be relevant for limb development. For instance, the important limb developmental regulator *Lmx1b *gene is surrounded by several intergenic transcripts, in particular by a well-described bi-directional transcript along its promoter region ([26]; Figure 4A). The hybridization pattern of this ncRNA showed a nearly identical spatial distribution, in dorsal limb mesenchyme, and its expression dynamics closely matched that of *Lmx1b*. 60 kb upstream of *Lmx1b*, we found a differentially expressed transcript, which may as well be an alternative start site for this gene since it is transcribed from the same DNA strand and expressed in an identical dorsal mesenchyme domain, even though it was more dramatically down-regulated at E15.5 than *Lmx1b*, as validated by qPCR. This example illustrates the difficulty to discriminate between genuine ncRNAs and parts of known transcription units located nearby.

Two bidirectional non-coding transcripts were also examined in some details by *in situ *hybridization, one starting very close from the start site of the *Hand2 *gene, whereas the other one starts at approximately 5 kb from the *Prdm16 *TSS (Figure 4B, D). Here again, expression patterns were nearly identical between the ncRNAs and their 'host' genes. Both *Hand2 *and the associated ncRNA were expressed in a patch on the posterior side of the developing limb bud, whereas the *Prdm16 *pair was transcribed strongly in distal autopod cells, in particular in growing digits. In both cases, it is thus likely that the transcription of these ncRNAs was achieved by regulatory modules used to control the genes located nearby (or *vice-versa*).

The fourth case selected as an example is at the *Sox4 *locus. *Sox4 *is a rather small gene, located on chromosome 13 and surrounded by large gene deserts, which display series of significant transcript signals during limb development. Several such peaks were observed both in 5' of the gene, where they matched small bi-directional transcripts (not shown), and in 3' where up to more than 400 kb large unspliced ncRNAs were scored (annotated). We focused our attention on a peak of transcriptional activity located approximately 50 kb downstream from the *Sox4 *3' UTR and tested it *by in situ *hybridization (Figure 4C). Again, the expression pattern was globally that of *Sox4*, though with some differences, in particular in the developing major body axis. Expression patterns in the limbs were similar to one another, suggesting once more that this ncRNA was under the control of the regulations acting over *Sox4*.

ncRNAs appear to be involved in diverse biological processes, as exemplified by the association of some lncRNAs with gene silencing (*e.g. *[8,27]). Our results indicate that these RNA species are frequently coupled either to actively transcribed regions, or to enhancer regions controlling genes of importance for developmental processes [28,29]. Accordingly, it is often unclear as to whether these transcripts result from a by-stander effect, *i.e. *by recruiting the activity of potent enhancer sequences located nearby, or if their control sequences evolved partly or entirely due to their own functional outcomes.

### Clustered genes and intergenic transcription

Gene clusters usually comprise structurally and/or functionally related genes that may have arisen *via *ancestral gene duplication events. Clustered genes often share some of their regulatory controls, either due to the existence of global regulatory modules controlling several genes at once, or because duplications also included some target sequences for upstream factors. On the other hand, gene duplication events certainly favored some functional divergence. For example, a duplication event generated the *Gdf10/Gdf2 *pair of transcription factors on chromosome 14; while *Gdf10 *is differentially expressed in the developing limb (up-regulated from E11.5 toward E13.5), *Gdf2 *is not expressed there. In contrast, both the *Zic2 *and *Zic5 *genes, also produced by a late duplication, are differentially expressed with the same dynamics (down-regulated from E11.5 toward E13.5), whereas the paralogous genes *Zic1 *and *Zic4 *were scored as non-expressed. Another case is the *Iroquois *gene family, with both the *Irx3/Irx5 *and *Irx1/Irx2 *pairs scored as being expressed, whereas the distal genes *Irx4 *and *Irx6 *were not detected. Interestingly, in the cases of *Zic5*, *Irx2 *and *Irx5*, promoter-associated bi-directional transcripts were also detected (*AK011684, AK086211 *and *AK017076*, respectively). Other examples for clustered expression of transcription factors are the *Myf5/6 *muscle-specific genes [30], which were up-regulated during differentiation, or the *Cbx2/8/4 *triplet, where *Cbx2 *was down-regulated in older limb buds, whereas *Cbx8 *and *Cbx4 *were expressed steadily throughout the three stages.

The four *Hox *gene clusters have been used as models for the coordinated regulation of expression in a variety of derivatives, including in developing limbs. In particular, the *HoxA *and *HoxD *clusters, which contain 11 and 9 genes, respectively, have essential functions during both hind- and forelimb development [31]. In both gene clusters, we observed transcription profiles in agreement with published datasets, showing a global decrease in intensity in E15.5 limb buds, mostly reflecting the cell type specialization of gene expression and hence a severe dilution effect when compared to the general expression in mesenchymal cells observed in E11.5. Extensive intergenic transcription was scored, whose expression profiles followed those of the surrounding *Hox *genes (Additional file 7). In the case of *HoxD*, three such intergenic regions encoded by the same strand as all *Hoxd *genes were tested by *in situ *hybridization. They all showed expression patterns virtually identical to those of the closest *Hoxd *genes, suggesting that they may be expanded 3' UTRs (additional file 7). In the case of *Hoxa13*, a bi-directional transcript was also scored, yet its expression profile was not related to that of *Hoxa13*. Recently, this transcript (*AK033508*) was named HOTTIP and shown to be involved in the regulation of *HoxA *cluster genes in cultured cells [32].

Finally, the largest gene clusters differentially expressed in developing limbs were associated with the skin developmental programs, including the keratin gene complexes on chromosome 11 and 15, or the *Sprrb *and *S100a *genes on chromosome 3, with a dominant expression at E15.5. These latter genes are part of the 'epidermal differentiation complex', spanning nearly 3 Mb and composed of members of different gene families clustered together [33-35]; (data not shown).

### Imprinted gene clusters and associated ncRNAs

More than 100 imprinted genes have been reported in the human genome, organized into distinct and large chromosomal regions http://www.har.mrc.ac.uk/research/genomic_imprinting/[36]. Imprinted gene clusters usually involve genes unrelated in structure or function, and in many cases they contain one or more ncRNAs that may regulate gene expression in *cis *(long ncRNAs) or in *trans *(miRNAs, C/D small nucleolar RNAs). A well-known example of a complex imprinted gene cluster is the *Dlk1-Gtl2 *locus on chromosome 12. The whole gene array located on the positive DNA strand seems to be up-regulated in limbs between day 11.5 and 13.5, with the most significant change observed for *Gtl2 *(*Meg3*) and the *Rian *non-coding RNAs (Additional file 7). *Dio3*, lying at the very 3' end of the complex was silent (not shown). The miRNA cluster, embedded within and in 5' outside the non-coding RNA *Mirg*, also gave a positive signal, suggesting they may be part of a larger transcript. Amongst the gene members of the cluster, *Gtl2 *was shown to be important during embryonic development [37], as deletion of the maternal allele resulted in perinatal lethality and skeletal muscle defects [38].

Other imprinted genes relevant for limb and/or bone development were found expressed, including the *Gnas-Nespas *locus, important for cartilage development [39] or *Cdkn1c*, within the *Kcnq1 *imprinted cluster [40], as well as the *Igf2-H19 *cluster, necessary for skeletal development and ossification [41,42]. *Plagl1 *(*Zac1*), a gene encoding a zinc-finger transcription factor, which regulates other imprinted genes and is also required for bone ossification [43], was progressively up-regulated, indicating the potential importance of imprinted genes during limb development.

## Conclusions

These datasets provide a valuable resource, either for research projects involving gene expression and regulation in developing mouse limbs, or as examples of tissue-specific, genome-wide transcriptional activities. More specifically, we found that expression signals are frequently confined to known transcript boundaries but tend to extend past the 3'ends of genes. Also, about 40 percent of known genes were found expressed in limbs of 11.5, 13.5 and 15.5 days old foetuses. We observed extensive intronic transcription, since about 50 percent of expressed genes also contained signals covering intronic sequences. After filtering, more than 4,000 intergenic regions remained clearly transcribed during the three developmental stages. Finally, we identified 49 long ncRNAs that were differentially expressed in our samples.

## Methods

### Sampling and microarray hybridization

C57Bl/6J embryonic forelimbs at stages 11.5 (20 limbs), 13.5 (8 limbs) and 15.5 (2 limbs) were dissected along the body wall and kept in RNAlater. Total RNA was extracted using RNeasy-mini columns (Qiagen), DNaseI digested on-column, treated with RNase free DNase I once more (Roche), and further processed according to the Affymetrix GeneChip WT Double-Stranded Target Assay manual. Briefly, ribosomal RNAs were depleted and samples were reverse transcribed. These cDNAs served as templates for cRNA synthesis (amplification), which were then reverse transcribed again. The products were fragmented, labeled and hybridized onto Genechip Mouse Tiling 1.1R arrays. The qualities of all initial steps were monitored by Bioanalyzer 2100 (Agilent). All microarray-related experiments were done in duplicate.

All experiences involving animals were carried according to the Swiss law, under the authorization number 1008/3482/0 (to DD).

### Microarray data processing

The tiling array set consisted of 14 chips. For each of these chips and each of the six samples (3 time-points × 2 duplicates), standard background correction and quantile normalization was performed; mismatch probes were not used. The correlation of probe intensities (log2) on all replicated arrays ranged form 0.84 and 0.91. Probe coordinates were converted to the July 2007 build of the mouse genome using the UCSC liftOver software http://hgdownload.cse.ucsc.edu/admin/exe/. For each chromosome on a chip, a summary procedure was then performed as follows. Known gene annotations were downloaded from UCSC http://genome.ucsc.edu/cgi-bin/hgTables and complemented by miRNA, snRNA and snoRNA annotations from ensemble http://www.ensembl.org. All probes in an exon were considered as a single block. In introns and intergenic regions, blocks were defined using a sliding window of 500 bp width. For each block, a linear model identical to that in the RMA method [44] was used to estimate the three condition-specific expression levels from the six samples. This regression was done after log transformation of the normalized intensity data. Thresholds for expression of exons were defined by the 99th percentile of the distribution of expression levels in the regions annotated as intergenic. Transcripts with 80% or more expressed exons were considered to be expressed. Genes with 50% or more expressed exons were considered to be expressed. Small RNAs were only considered if they were larger than 100 bp.

### Identification of differentially expressed genes

Differences in expression were calculated by taking the median (log-scale) expression level across the exons of a gene, and then calculating the differences between pairs of experimental conditions. A permutation method was used to define significance thresholds on differential expression: probes were permutated locally along the genome as follows: a random variable drawn from a Gaussian distribution (standard deviation = 1 Mb) for each probe and was added to the probe coordinates. The probes were then ranked according to their new coordinates. In the permutated dataset, the intensity of a probe was then replaced by the intensity of the probe with equal rank in the randomized coordinates. Five permutated datasets were generated and differential expression levels of genes were calculated as above. Expression differences greater than the 99th percentile or smaller than the first percentile of the expression differences in the combined permutated datasets were considered as significant.

### Intronic and intergenic signal analyses

Intronic signals were defined as continuous regions above threshold of at least 300 bp length and containing no unspotted regions (regions without hybridization probes) of more than 100 bp. Intergenic signals were defined as continuous regions above threshold of at least 300 bp lengths. Once identified, intergenic signals were classified as flanking UCSC annotations if they were within 100 bp of a UCSC known gene annotation. Wherever possible, intergenic signals not classified as flanking UCSC annotations were identified from Ensembl annotation, differentiating between Ensembl overlap, where an overlap of at least 200 bp existed between an intergenic signal and an Ensembl annotation, and Ensembl flanking, where the intergenic signal was within 100 bp of the Ensembl annotation. The remaining signals were considered and termed unexplained. expressed intergenic regions (orange) at E11.5 were annotated as 'close to' an expressed gene (red), 'close to' a non-expressed (blue), 'not close to' a gene (green). For Figure 3, the distance cut-off to assign 'close' varied form 1 Kb to 1 Mb (x-axis) using bin size increasing by 1 Kb from 1 Kb to 20 Kb, by 10 Kb from 20 Kb to 100 Kb and by 100 Kb from 100 Kb to 1 Mb.

### Conservation of ncRNA sequences

PhastCons conservation scores [45] were downloaded from UCSC website using 29 vertebrate genomes alignments with the Mouse (mm9-PhasCons30way). Mean conservation scores were calculated for genomic regions covering the selected ncRNAs.

### Whole mount in situ hybridization and quantitative PCR

Whole mount *in situ *hybridizations were performed according to standard protocol. Amplified cDNA products were ligated into pTopo-BluntII (Invitrogen) vector. Digoxigenin (Roche) labeled riboprobes were produced using T7 or Sp6 RNA polymerase (Promega). Hybridizations were carried out overnight at 68°C. Hybridizations were detected using NBT/BCIP solutions (Roche). Real-time qPCRs were done in triplicate with Express Sybr GreenER premix (Invitrogen) using CFX96 Real-Time system (Biorad). Primers are compiled in the supplementary material (Additional file 8).

### Data availability

Raw tiling array data (CEL files) as well as .wig files suitable for display in the UCSC genome browser are available at the Gene Expression Omnibus (GEO) under accession number: GSE27417 http://www.ncbi.nlm.nih.gov/geo/query/acc.cgi?acc=GSE27417.

## Abbreviations

AER: Apical Ectodermal Ridge; AP: Anterior to Posterior; DV: Dorsal to Ventral; ncRNAs: Non-coding RNAs; lncRNAs: Long non-coding RNAs; TSS: Transcription start site; UTR: Untranslated region; TF: Transcription factor.

## Competing interests

The authors declare that they have no competing interests.

## Authors' contributions

IG helped design and carried out the experiments and drafted the manuscript, BS analyzed the data, FN supervised data analysis and interpretation and DD designed the experiments and wrote the paper. All authors helped writing the article.

## Supplementary Material

Additional file 1 **Supplementary Table 1: List of identified differentially expressed genes**. Plus 1 indicate up-regulation, minus 1 down-regulation, zero means no change, respectively. The 2 comparisons are shown in parallel columns (*i.e. *E13.5 minus E11.5 and E15.5 minus E13.5). Click here for file

Additional file 2 **Supplementary Figure 1: Validation of some selected differentially expressed genes by quantitative PCR and/or whole mount *in situ *hybridization**. **A**. Bar charts show relative gene expression levels, with the measured transcript level at E11.5 taken as a reference ('1'). A: Relative expression for *Hoxd8 *to *Hoxd13*; *Hoxc6*; *Cbln1*; *Lhx9 *and *Aff3*. **B: **Relative gene expression of bone specific marker genes that were up-regulated at E15.5: *Col1a1*; *Ibsp*; *Spp1*. **C: **Relative gene expression of *Col2a1*; *Aldh1a2*; *Osterix (Sp7)*; *Noggin *and *Tmem8c*. Steady-state levels of selected RNAs were assessed at the three stages (in green for E11.5; blue for E13.5 and red for E15.5). While expression of control *Hoxd *genes expectedly decreased at E15.5 (probably due to the decrease of mesenchymal cell mass at this stage), expression of genes specific for late stages of cartilage differentiation was markedly increased. **D: ***In situ *hybridizations of selected differentially expressed genes. Genes were selected according to their various expression dynamics: *Lhx9 *and *Aff3 *were progressively down-regulated during development (*i.e. *from E11.5 to E15.5); *Tmem8c *was progressively up-regulated; *Noggin *tend to up-regulated between E11.5 and E13.5 but down-regulated later (see qPCR data above) while *Hoxc6 *and *Cbln1 *were expressed only at E11.5 then rapidly down-regulated. Gene symbols and developmental stages are indicated. Click here for file

Additional file 3 **Supplementary Figure 2: Gene ontology analysis of differentially expressed genes using GOMiner**. Hierarchical clustering of enriched gene ontology terms (biological functions) characterizing differentially expressed genes. The settings were: evidence level of 3, minimum cluster size of 5 and maximum FDR of 0.05. **A, B**. Clusters of enriched GO terms for those genes differentially expressed, either between E13.5 and E11.5 (**A**) or between E15.5 and 13.5 **(B)**. X-axis indicates GO categories, Y-axis for differentially expressed genes. Red areas indicate up-regulated genes, whereas the green areas represent down-regulated genes those are sharing the same functional categories. Click here for file

Additional file 4 **Supplementary Table 2: Top 50 cluster categories ranked by p-values (calculated from Fisher's exact test) and GO term enrichments (minimum 2 fold)**. The left panel shows the enriched categories for those genes that are differentially expressed between E11.5 and E13.5, whereas the panel in the right is for those genes differentially expressed between E13.5 and E15.5. Click here for file

Additional file 5 **Supplementary Table 3: List of differentially expressed genes that were previously associated with short or abnormal limb phenotype, based on Mammalian Phenotype Ontology**. Click here for file

Additional file 6 **Supplementary Table 4: Gene model categories of intergenic transcripts identified via Ensembl**. All intergenic regions were screened for potential overlaps with Ensembl annotation. Identified genes were classified according to Ensembl gene model categories. Click here for file

Additional file 7 **Supplementary Figure 3: Intergenic transcription within gene clusters. A**. Tiling array gene expression data along the entire *HoxD *gene cluster on chromosome 2. Transcription is scored outside annotated transcription units, at least in three regions; *igs1*, *igs2 *and *igs3 *(black bars). **B: ***In situ *hybridizations on E12.5 embryos, with three different probes corresponding to these transcribed intergenic regions. igs1 lies between *Hoxd11 *and *Hoxd12*, whereas both igs2 and igs3 are located between *Hoxd10 *and *Hoxd11*. The transcription profiles of these RNAs follow the general logic of the gene cluster and thus resemble the expression of the neighboring genes. **C**. Tiling array expression data along the entire *HoxA *gene cluster on chromosome 6, showing intergenic transcription, also from the opposite DNA strand (*Hoxa11as *and *AK033508*). **D**. Transcription profiles at the *Dlk1-Gtl2 *imprinted gene cluster on chromosome 12. The vertical black bars (on the 'positive' strand) point either to C/D small RNAs, mapping around *Rtl1*, or to miRNAs located around *Mirg*. The small transcript overlapping with *Rtl1 *is referred to as *Rtl1*- *antisense *transcript. All scales on tiling array's Y-axes are in log_2_. Click here for file

Additional file 8 **Supplementary Table 5: List of oligonucleotide sequences used for quantitative PCR**. (A) or cloning procedures (B). All sequences are indicated from 5-prime to 3-prime. Click here for file
