# Supplementary figures and images for: Analysis of the dynamics of limb transcriptomes during mouse development
Source: BMC Dev Biol. 2011 Jul 29;11:47. doi: 10.1186/1471-213X-11-47 (PMC3160909; doi:10.1186/1471-213X-11-47)

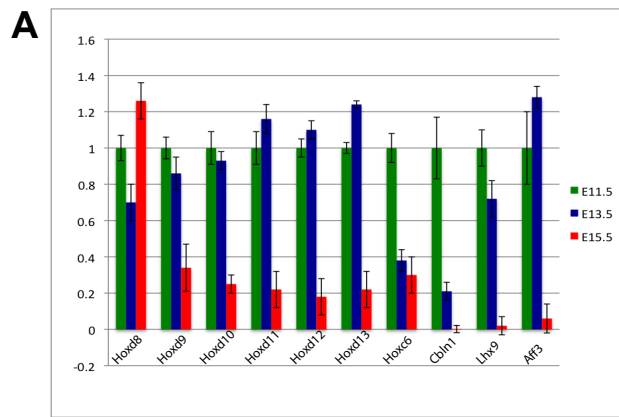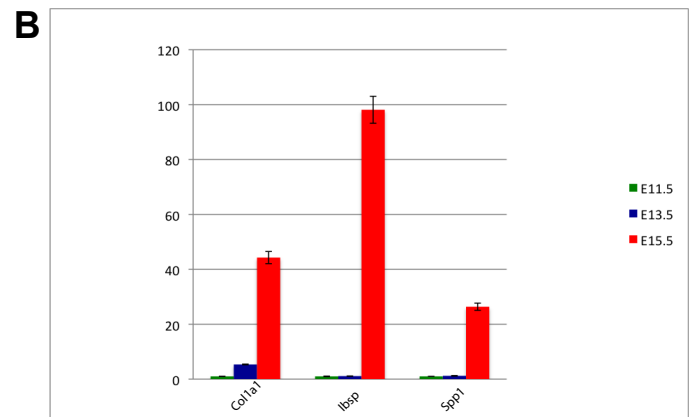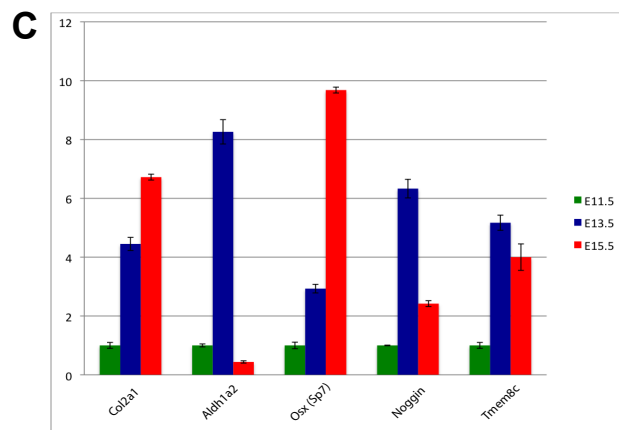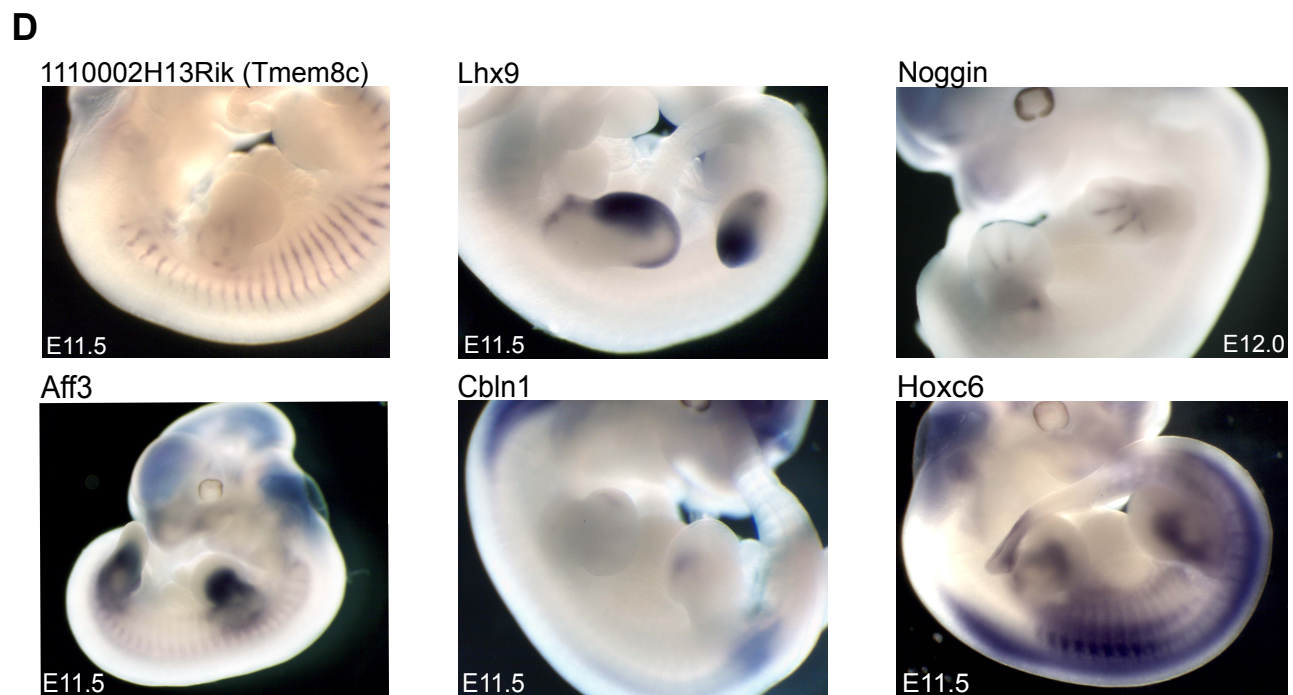

Supplement: Additional file 2 — Supplementary Figure 1: Validation of some selected differentially expressed genes by quantitative PCR and/or whole mount in situ hybridization. A. Bar charts show relative gene expression levels, with the measured transcript level at E11.5 taken as a reference ('1'). A: Relative expression for Hoxd8 to Hoxd13; Hoxc6; Cbln1; Lhx9 and Aff3. B: Relative gene expression of bone specific marker genes that were up-regulated at E15.5: Col1a1; Ibsp; Spp1. C: Relative gene expression of Col2a1; Aldh1a2; Osterix (Sp7); Noggin and Tmem8c. Steady-state levels of selected RNAs were assessed at the three stages (in green for E11.5; blue for E13.5 and red for E15.5). While expression of control Hoxd genes expectedly decreased at E15.5 (probably due to the decrease of mesenchymal cell mass at this stage), expression of genes specific for late stages of cartilage differentiation was markedly increased. D: In situ hybridizations of selected differentially expressed genes. Genes were selected according to their various expression dynamics: Lhx9 and Aff3 were progressively down-regulated during development (i.e. from E11.5 to E15.5); Tmem8c was progressively up-regulated; Noggin tend to up-regulated between E11.5 and E13.5 but down-regulated later (see qPCR data above) while Hoxc6 and Cbln1 were expressed only at E11.5 then rapidly down-regulated. Gene symbols and developmental stages are indicated. [file 1471-213X-11-47-S2.PDF]

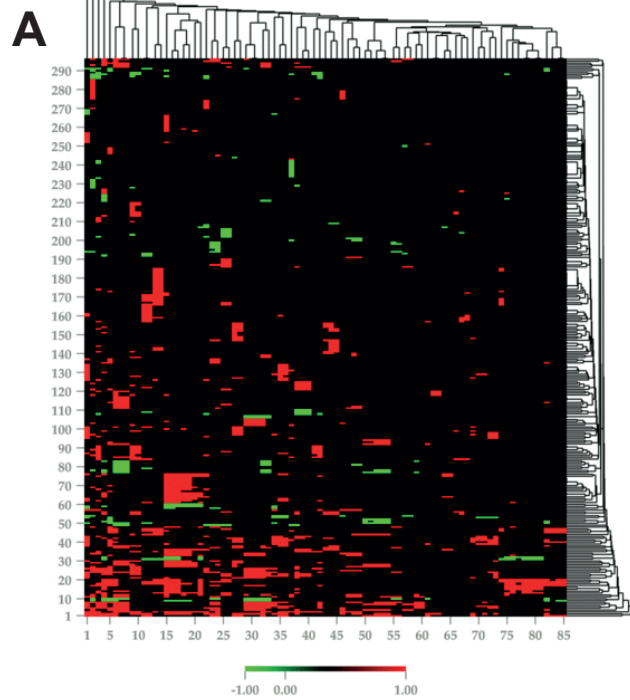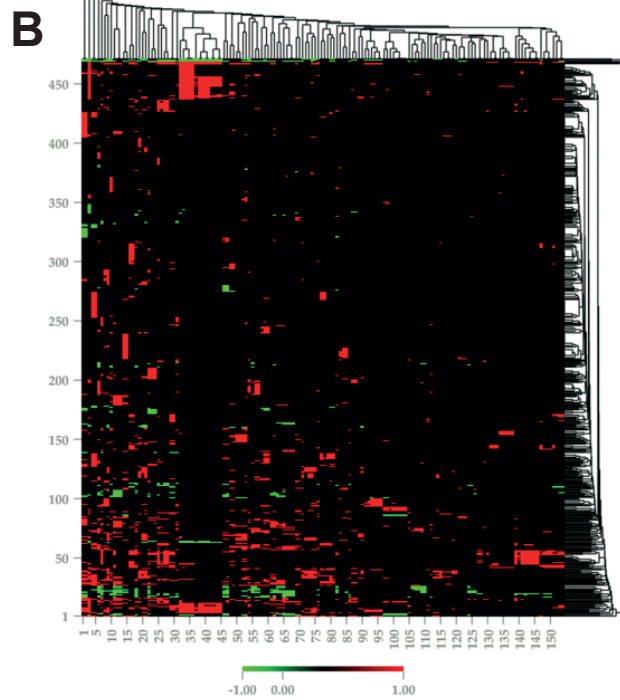

Supplement: Additional file 3 — Supplementary Figure 2: Gene ontology analysis of differentially expressed genes using GOMiner. Hierarchical clustering of enriched gene ontology terms (biological functions) characterizing differentially expressed genes. The settings were: evidence level of 3, minimum cluster size of 5 and maximum FDR of 0.05. A, B. Clusters of enriched GO terms for those genes differentially expressed, either between E13.5 and E11.5 (A) or between E15.5 and 13.5 (B). X-axis indicates GO categories, Y-axis for differentially expressed genes. Red areas indicate up-regulated genes, whereas the green areas represent down-regulated genes those are sharing the same functional categories. [file 1471-213X-11-47-S3.PDF]

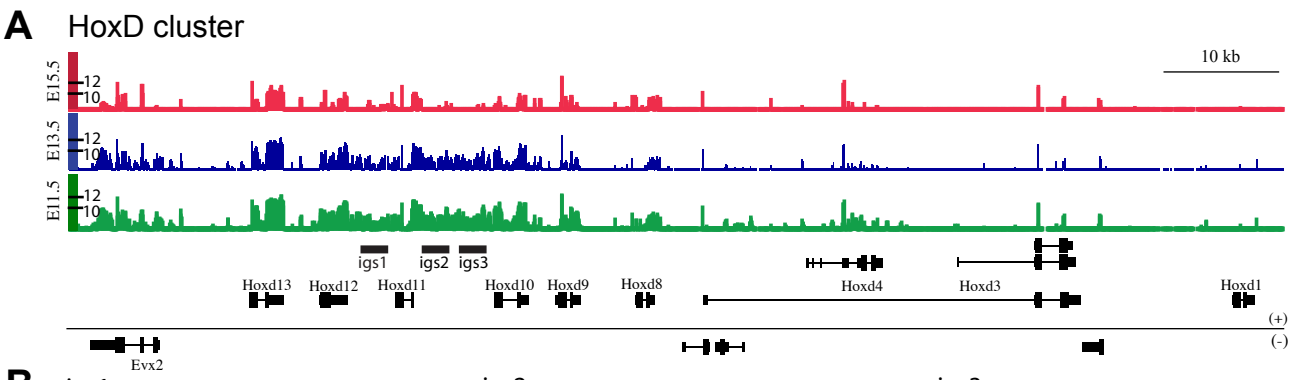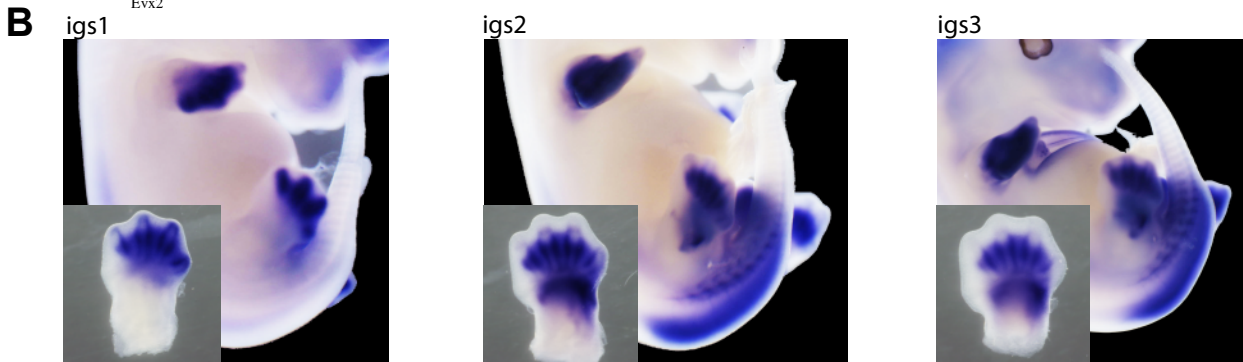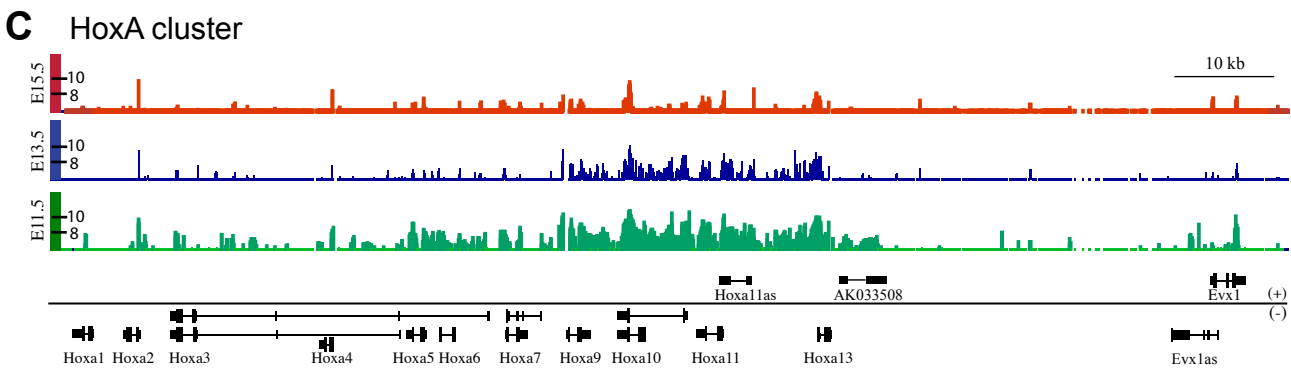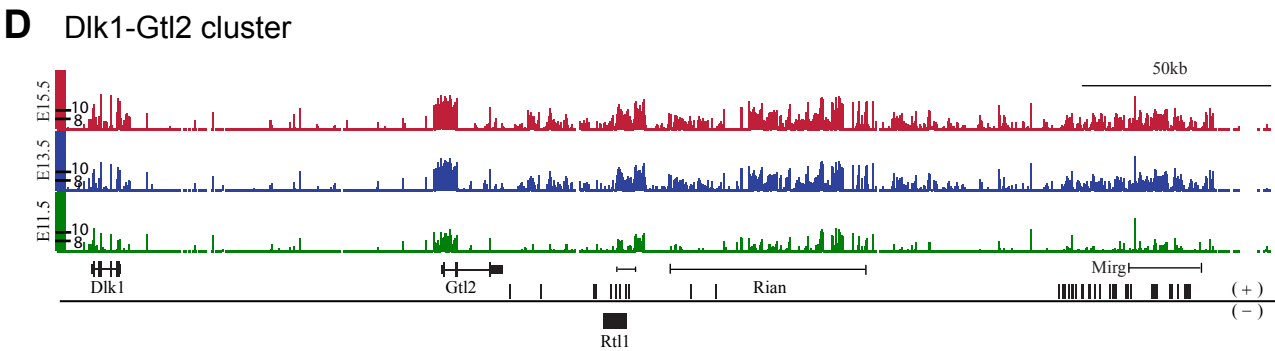

Supplement: Additional file 7 — Supplementary Figure 3: Intergenic transcription within gene clusters. A. Tiling array gene expression data along the entire HoxD gene cluster on chromosome 2. Transcription is scored outside annotated transcription units, at least in three regions; igs1, igs2 and igs3 (black bars). B: In situ hybridizations on E12.5 embryos, with three different probes corresponding to these transcribed intergenic regions. igs1 lies between Hoxd11 and Hoxd12, whereas both igs2 and igs3 are located between Hoxd10 and Hoxd11. The transcription profiles of these RNAs follow the general logic of the gene cluster and thus resemble the expression of the neighboring genes. C. Tiling array expression data along the entire HoxA gene cluster on chromosome 6, showing intergenic transcription, also from the opposite DNA strand (Hoxa11as and AK033508). D. Transcription profiles at the Dlk1-Gtl2 imprinted gene cluster on chromosome 12. The vertical black bars (on the 'positive' strand) point either to C/D small RNAs, mapping around Rtl1, or to miRNAs located around Mirg. The small transcript overlapping with Rtl1 is referred to as Rtl1- antisense transcript. All scales on tiling array's Y-axes are in log2. [file 1471-213X-11-47-S7.PDF]
