# Supplementary material for: Analysis of the dynamics of limb transcriptomes during mouse development
Source: BMC Dev Biol. 2011 Jul 29;11:47. doi: 10.1186/1471-213X-11-47 (PMC3160909; doi:10.1186/1471-213X-11-47)
Supplement: Additional file 4 — Supplementary Table 2: Top 50 cluster categories ranked by p-values (calculated from Fisher's exact test) and GO term enrichments (minimum 2 fold). The left panel shows the enriched categories for those genes that are differentially expressed between E11.5 and E13.5, whereas the panel in the right is for those genes differentially expressed between E13.5 and E15.5. [file 1471-213X-11-47-S4.PDF]

Table S2. Top 50 cluster categories ranked by p-values (calculated from Fisher's exact test) and GO term enrichments (minimum 2 fold). The left panel shows the enriched categories for those genes that are differentially expressed between E11.5 and E13.5, whereas the panel in the right is for those genes differentially expressed between E13.5 and E15.5.

GO categories - E13.5 vs. E11.5	Enrichment	Log10 (p-value)
GO:0007517_muscle_organ_development	2.415	-11.2042
GO:0003012_muscle_system_process	2.650	-7.5219
GO:0006936_muscle_contraction	2.617	-6.7868
GO:0060538_skeletal_muscle_organ_development	2.506	-6.3100
GO:0014706_striated_muscle_tissue_development	2.194	-6.0609
GO:0060537_muscle_tissue_development	2.159	-5.9985
GO:0060541_respiratory_system_development	2.676	-5.8222
GO:0030324_lung_development	2.847	-5.7294
GO:0030323_respiratory_tube_development	2.772	-5.5059
GO:0001822_kidney_development	2.443	-5.3562
GO:0001655_urogenital_system_development	2.239	-5.2995
GO:0007519_skeletal_muscle_tissue_development	2.372	-5.2495
GO:0007263_nitric_oxide_mediated_signal_transduction	6.108	-5.1942
GO:0042060_wound_healing	2.309	-5.1561
GO:0030198_extracellular_matrix_organization	2.587	-5.1340
GO:0030199_collagen_fibril_organization	4.199	-4.8028
GO:0060348_bone_development	2.164	-4.5586
GO:0060571_morphogenesis_of_an_epithelial_fold	5.235	-4.4983
GO:0043062_extracellular_structure_organization	2.142	-4.3395
GO:0050679_positive_regulation_of_epithelial_cell_proliferation	3.546	-4.2925
GO:0003013_circulatory_system_process	2.036	-4.2594
GO:0008015_blood_circulation	2.036	-4.2594
GO:0048589_developmental_growth	2.268	-4.2482
GO:0042692_muscle_cell_differentiation	2.245	-4.1721
GO:0055008_cardiac_muscle_tissue_morphogenesis	3.817	-3.9786
GO:0060415_muscle_tissue_morphogenesis	3.817	-3.9786
GO:0001503_ossification	2.114	-3.9733
GO:0003007_heart_morphogenesis	2.346	-3.8839
GO:0002062_chondrocyte_differentiation	3.475	-3.8836
GO:0050673_epithelial_cell_proliferation	2.452	-3.8388
GO:0050678_regulation_of_epithelial_cell_proliferation	2.553	-3.7249
GO:0045214_sarcomere_organization	5.497	-3.6280
GO:0006182_cGMP_biosynthetic_process	6.544	-3.5763
GO:0055010_ventricular_cardiac_muscle_morphogenesis	4.072	-3.5082
GO:0060349_bone_morphogenesis	3.585	-3.3846
GO:0010927_cellular_component_assembly_involved_in_morphogenesis	2.770	-3.3473
GO:0006941_striated_muscle_contraction	2.893	-3.3219
GO:0042246_tissue_regeneration	3.054	-3.3132
GO:0048762_mesenchymal_cell_differentiation	2.360	-3.2808
GO:0060485_mesenchyme_development	2.360	-3.2808
GO:0030239_myofibril_assembly	3.436	-3.2240
GO:0042634_regulation_of_hair_cycle	7.330	-3.1924
GO:0045663_positive_regulation_of_myoblast_differentiation	7.330	-3.1924
GO:0051797_regulation_of_hair_follicle_development	7.330	-3.1924
GO:0050819_negative_regulation_of_coagulation	5.726	-3.1912
GO:0051149_positive_regulation_of_muscle_cell_differentiation	5.726	-3.1912
GO:0060572_morphogenesis_of_an_epithelial_bud	5.726	-3.1912
GO:0001656_metanephros_development	2.748	-3.0963
GO:0060350_endochondral_bone_morphogenesis	4.008	-3.0746
GO:0031589_cell-substrate_adhesion	2.106	-3.0649

GO categories - E15.5 vs. 13.5	Enrichment	Log10 (p-value)
GO:0003012_muscle_system_process	3.049	-14.3351
GO:0006936_muscle_contraction	3.041	-13.2093
GO:0009611_response_to_wounding	2.202	-12.3502
GO:0008544_epidermis_development	2.756	-11.0730
GO:0007398_ectoderm_development	2.600	-10.2342
GO:0030198_extracellular_matrix_organization	3.005	-9.8558
GO:0030199_collagen_fibril_organization	4.731	-8.2922
GO:0002526_acute_inflammatory_response	4.120	-7.8684
GO:0006941_striated_muscle_contraction	3.548	-6.8604
GO:0043062_extracellular_structure_organization	2.289	-6.8286
GO:0042060_wound_healing	2.266	-6.3884
GO:0030239_myofibril_assembly	4.139	-6.2666
GO:0006954_inflammatory_response	2.196	-6.1483
GO:0048771_tissue_remodeling	2.365	-6.0973
GO:0001655_urogenital_system_development	2.102	-5.7462
GO:0031032_actomyosin_structure_organization	3.821	-5.6940
GO:0018149_peptide_cross-linking	4.258	-5.6199
GO:0055002_striated_muscle_cell_development	3.548	-5.5290
GO:0001822_kidney_development	2.230	-5.4524
GO:0003013_circulatory_system_process	2.020	-5.3867
GO:0008015_blood_circulation	2.020	-5.3867
GO:0032101_regulation_of_response_to_external_stimulus	2.425	-5.3271
GO:0060048_cardiac_muscle_contraction	4.055	-5.3112
GO:0001503_ossification	2.122	-5.2021
GO:0060348_bone_development	2.067	-5.1688
GO:0050727_regulation_of_inflammatory_response	3.093	-5.1573
GO:0010927_cellular_component_assembly_involved_in_morphogenesis	2.970	-5.1251
GO:0030216_keratinocyte_differentiation	3.326	-5.0870
GO:0009913_epidermal_cell_differentiation	2.903	-4.9570
GO:0031347_regulation_of_defense_response	2.473	-4.7808
GO:0055001_muscle_cell_development	3.130	-4.6908
GO:0006953_acute-phase_response	5.519	-4.5243
GO:0055010_ventricular_cardiac_muscle_morphogenesis	3.942	-4.3597
GO:0006937_regulation_of_muscle_contraction	2.741	-4.3294
GO:0045785_positive_regulation_of_cell_adhesion	2.741	-4.3294
GO:0055003_cardiac_myofibril_assembly	6.082	-4.3239
GO:0030155_regulation_of_cell_adhesion	2.152	-4.2627
GO:0002062_chondrocyte_differentiation	3.181	-4.2239
GO:0060350_endochondral_bone_morphogenesis	3.991	-4.0244
GO:0051146_striated_muscle_cell_differentiation	2.566	-3.9081
GO:0022612_gland_morphogenesis	2.176	-3.7940
GO:0055008_cardiac_muscle_tissue_morphogenesis	3.252	-3.7568
GO:0060415_muscle_tissue_morphogenesis	3.252	-3.7568
GO:0032330_regulation_of_chondrocyte_differentiation	4.516	-3.6748
GO:0051241_negative_regulation_of_multicellular_organismal_process	2.119	-3.6046
GO:0031349_positive_regulation_of_defense_response	2.795	-3.5339
GO:0034754_cellular_hormone_metabolic_process	2.795	-3.5339
GO:0051216_cartilage_development	2.092	-3.5134
GO:0031960_response_to_corticosteroid_stimulus	2.217	-3.4928
GO:0050818_regulation_of_coagulation	3.784	-3.4141
